# Fungal Tracheobronchitis in Lung Transplant Recipients: Incidence and Utility of Diagnostic Markers

**DOI:** 10.3390/jof9010003

**Published:** 2022-12-20

**Authors:** Helena Hammarström, Jesper Magnusson, Anna Stjärne Aspelund, Jakob Stenmark, Jenny Isaksson, Nahid Kondori, Gerdt C Riise, Christine Wennerås, Vanda Friman

**Affiliations:** 1 Department of Infectious Diseases, Institute of Biomedicine, Sahlgrenska Academy, University of Gothenburg, 416 85 Gothenburg, Sweden; 2 Department of Infectious Diseases, Region Västra Götaland, Sahlgrenska University Hospital, 416 85 Gothenburg, Sweden; 3 Department of Respiratory Medicine and Allergology, Institute of Internal Medicine, Sahlgrenska Academy, University of Gothenburg, 416 85 Gothenburg, Sweden; 4 Department of Clinical Sciences, Division of Infection Medicine, University of Lund, 222 10 Lund, Sweden; 5 Department of Clinical Microbiology and Infection Prevention and Control, Skåne University Hospital, 222 10 Lund, Sweden; 6 Department of Clinical Microbiology, Region Västra Götaland, Sahlgrenska University Hospital, 416 85 Gothenburg, Sweden

**Keywords:** fungal infection, fungal tracheobronchitis, lung transplant recipients, betaglucan, galactomannan

## Abstract

Fungal tracheobronchitis caused by *Aspergillus* and *Candida* spp. is a recognized complication after lung transplantation, but knowledge of the incidence of *Candida* tracheobronchitis is lacking. The diagnosis relies on fungal cultures in bronchoalveolar lavage fluid (BALF), but cultures have low specificity. We aimed to evaluate the one-year incidence of fungal tracheobronchitis after lung transplantation and to assess the utility of diagnostic markers in serum and BALF to discriminate fungal tracheobronchitis from colonization. Ninety-seven consecutively included adult lung-transplant recipients were prospectively followed. BALF and serum samples were collected at 1, 3 and 12 months after transplantation and analyzed for betaglucan (serum and BALF), neutrophils (BALF) and galactomannan (BALF). Fungal tracheobronchitis was defined according to consensus criteria, modified to include *Candida* as a mycologic criterion. The cumulative one-year incidence of *Candida* and *Aspergillus* tracheobronchitis was 23% and 16%, respectively. Neutrophils of >75% of total leukocytes in BALF had 92% specificity for *Candida* tracheobronchitis. The area under the ROC curves for betaglucan and galactomannan in BALF to discriminate *Aspergillus* tracheobronchitis from colonization or no fungal infection were high (0.86 (*p* < 0.0001) and 0.93 (*p* < 0.0001), respectively). To conclude, the one-year incidence of fungal tracheobronchitis after lung transplantation was high and dominated by *Candida* spp. Diagnostic markers in BALF could be useful to discriminate fungal colonization from tracheobronchitis.

## 1. Introduction

Invasive fungal infections constitute a major cause of morbidity and mortality after lung transplantation [1,2]. In a large prospective multicenter study of solid-organ-transplant recipients, lung-transplant recipients had the second highest cumulative one-year incidence of overall invasive fungal infections (9%) [1], and in another study, the prevalence rate was 19 invasive fungal infections per 100 lung transplantations [3]. Fungal tracheobronchitis predominantly affects lung transplant recipients and is a recognized complication during the first year following lung transplantation [4,5]. Fungal tracheobronchitis is commonly caused by *Aspergillus* spp. [6,7], but *Candida* tracheobronchial infections, including infections localized to the anastomotic site, are also well-described [6,7,8,9]. Studies describing the epidemiology of fungal infections after lung transplantation rely on internationally established criteria for the definition of invasive fungal infections in immunosuppressed hosts and transplant recipients [10,11]. These guidelines include a definition for probable *Aspergillus* tracheobronchitis. However, despite being a recognized entity of fungal infection after lung transplantation, there is no established definition for probable *Candida* tracheobronchitis, implying that these infections are underreported. Knowledge of the incidence of fungal tracheobronchitis, including both *Aspergillus* and *Candida* spp., in this patient cohort is thus lacking.

The diagnosis of fungal tracheobronchitis is based on histopathologic evidence of fungal invasion of bronchial tissue, but due to limited sensitivity and pertinent difficulties in obtaining tissue specimens for histopathologic analysis [12], the diagnosis often relies solely on fungal cultures from the lower respiratory tract. However, the isolation of *Candida* and *Aspergillus* spp. from respiratory specimens of lung-transplant recipients is a common occurrence during the first postoperative year [13,14], and whether or not positive cultures reflect respiratory tract colonization or tissue-invasive tracheobronchial infection is not always easily determined.

A high neutrophil count in bronchoalveolar lavage fluid (BALF) from lung transplant recipients may be associated with lower respiratory tract infections [15,16], but whether or not neutrophils in BALF can help to distinguish fungal colonization from tissue invasive fungal tracheobronchial infections in this patient group has not been clearly demonstrated. Assays measuring the *Aspergillus* cell-wall component galactomannan in BALF are widely used and recommended tools for the diagnosis of invasive pulmonary aspergillosis [4,11], although specific data on tracheobronchial infections are limited. Detection of the pan fungal cell-wall component 1,3-β-D-glucan (betaglucan) in serum is another established diagnostic tool for invasive fungal infections, but its usefulness to diagnose fungal infections following lung transplantation seems limited [17]. The utility of measuring betaglucan levels in BALF was evaluated in a meta-analysis showing marginal accuracy for diagnosing invasive pulmonary fungal infections [18]. However, its value for distinguishing fungal tracheobronchitis from colonization after lung transplantation remains to be determined.

This prospective study aimed to assess the incidence of fungal tracheobronchitis during the first year after lung transplantation and to evaluate the utility of betaglucan in serum, and betaglucan, galactomannan and neutrophils in BALF as diagnostic tools for tracheobronchitis and as tools for discriminating tissue-invasive tracheobronchitis from fungal colonization.

## 2. Materials and Methods

All adult Swedish patients accepted for lung transplantation at Sahlgrenska University Hospital in Gothenburg between May 2012 and December 2014 who gave oral and written consent to participate were prospectively and consecutively enrolled in the study. The final study cohort included all study patients who were alive two weeks after transplantation. The Swedish national protocol for immunosuppression after lung transplantation was followed, which at the time consisted of induction therapy with anti-thymocyte globulin and maintenance therapy with cyclosporine or tacrolimus in a tapering dose combined with mycophenolate mofetil and prednisone. Oral nystatin was given as antifungal prophylaxis for four weeks. Patients with respiratory tract colonization by *Aspergillus* before transplantation were given four weeks of voriconazole or liposomal Amphotericin B treatment following transplantation. The study cohort was followed systematically during 12 months after transplantation, including clinical controls and bronchoscopies approximately at one, three and twelve months post-transplant and with additional bronchoscopies in response to clinical symptoms, according to the routine clinical follow-up protocol at the Sahlgrenska University Hospital Transplant Center. Clinical and laboratory data were recorded in an electronic case-report form, and serum and BALF samples were collected at all control episodes. Bronchoscopy procedures were recorded according to clinical routine. Transbronchial biopsies were collected at one and three months unless clinically contraindicated and at twelve months if clinically indicated. All biopsies were analyzed histopathologically for signs of acute rejection and for the presence of fungal elements.

Cell differential count; cultures for bacteria, yeast and mold; and direct microscopy for fungal elements were performed on BALF samples according to standard routines at the Sahlgrenska University Hospital’s Departments of Clinical Pathology and Clinical Microbiology, respectively. Galactomannan optical density indices (ODIs) were analyzed on BALF samples, using the Platelia™ Aspergillus Enzyme Immunoassay (Bio-Rad, Marnes-la-Coquette, France) according to the manufacturer’s instructions. The Glucatell assay kit (Associates of Cape Cod, Falmouth, MA, USA) was used for measuring levels of betaglucan in serum and BALF samples according to the manufacturer’s instruction, which was modified to include heat pretreatment of samples at 75 °C. BALF samples were centrifuged at 10,000 rpm for 10 min, and aliquots of the supernatant were pretreated and analyzed according to the protocol for serum samples. Betaglucan results were reported between the minimum and maximum levels of detection (50–400 pg/mL). All analyses were performed consecutively on fresh patient samples.

The classification of proven or probable fungal tracheobronchitis was performed retrospectively and was based on the standardized international criteria for fungal infections in lung-transplant recipients formulated by the International Society for Heart and Lung Transplantation (ISHLT) [10]. Patients with histologic evidence of fungal invasion of bronchial tissue were classified as proven cases. A probable case was defined based on mycologic evidence of fungi in BALF (i.e., fungal culture yielding a mold or a positive galactomannan index), along with compatible clinical symptoms of tracheobronchitis and typical endobronchial lesions (i.e., erythema, ulceration, necrosis and/or pseudomembrane formation). Prior to the case classification, all recorded bronchoscopies from study patients were reviewed by a senior transplant pulmonologist (J.M.). Endobronchial lesions were systematically classified according to the abovementioned criteria. Considering that international consensus criteria for fungal infections in lung-transplant recipients [10,19] lack a definition for *Candida* tracheobronchitis, we took the liberty to extend the ISHLT criteria for fungal tracheobronchitis to also include positive cultures for *Candida* spp. in BALF as a mycological criterium for probable *Candida* tracheobronchitis. The classification of fungal tracheobronchitis was performed with the researchers blinded to the results of betaglucan and neutrophil count in BALF. Fungal colonization was defined as a BALF culture yielding *Aspergillus* or *Candida* spp. without fulfilling the definition of proven or probable fungal tracheobronchitis or invasive pulmonary aspergillosis. Patients who were first classified as colonized patients but at a later bronchoscopy episode fulfilled the criteria of tracheobronchitis were classified as patients with tracheobronchitis only. Acute rejection was defined as a transbronchial biopsy showing a rejection grade of A1 or higher according to the International Society for Heart and Lung Transplantation [20].

The cumulative one-year incidence of fungal tracheobronchitis was calculated by using a survival analysis. Patients who died before the end of the one-year follow-up time were treated as censored subjects. A comparison of the betaglucan levels, galactomannan indices and neutrophil counts in patients with fungal tracheobronchitis and fungal colonization was performed by using the Mann–Whitney test of mean ranks. Receiver operating characteristics (ROCs) curves were performed to assess the overall ability of each diagnostic marker to discriminate (1) fungal tracheobronchitis from colonization and (2) fungal tracheobronchitis from colonization or no evidence of fungi. Analyses for *Candida* and *Aspergillus* were performed separately. Galactomannan was evaluated only for patients with *Aspergillus*, excluding those patients where galactomannan was the only mycological criterion for the classification of *Aspergillus* tracheobronchitis. For scenarios with an acceptable area under the ROC curve (≥75%), further analyses of sensitivity and specificity at different cutoff levels were performed. Cutoff levels were chosen based on the results of the ROC curve analyses. The per-patient values for each diagnostic marker selected for the abovementioned evaluation were as follows. For patients with tracheobronchitis, the sample collected on the same day as the first fulfillment of the criteria defining the episode of tracheobronchitis was selected. For patients with fungal colonization, the sample collected on the same day as the first episode of positive fungal culture was selected. For patients with no evidence of fungi, the sample collected at the clinical control performed one month after transplantation was selected since all patients were still hospitalized and the vast majority of patients had a control performed at this point. A *p*-value < 0.05 was considered statistically significant.

## 3. Results

### 3.1. Patients

**Figure 1:**
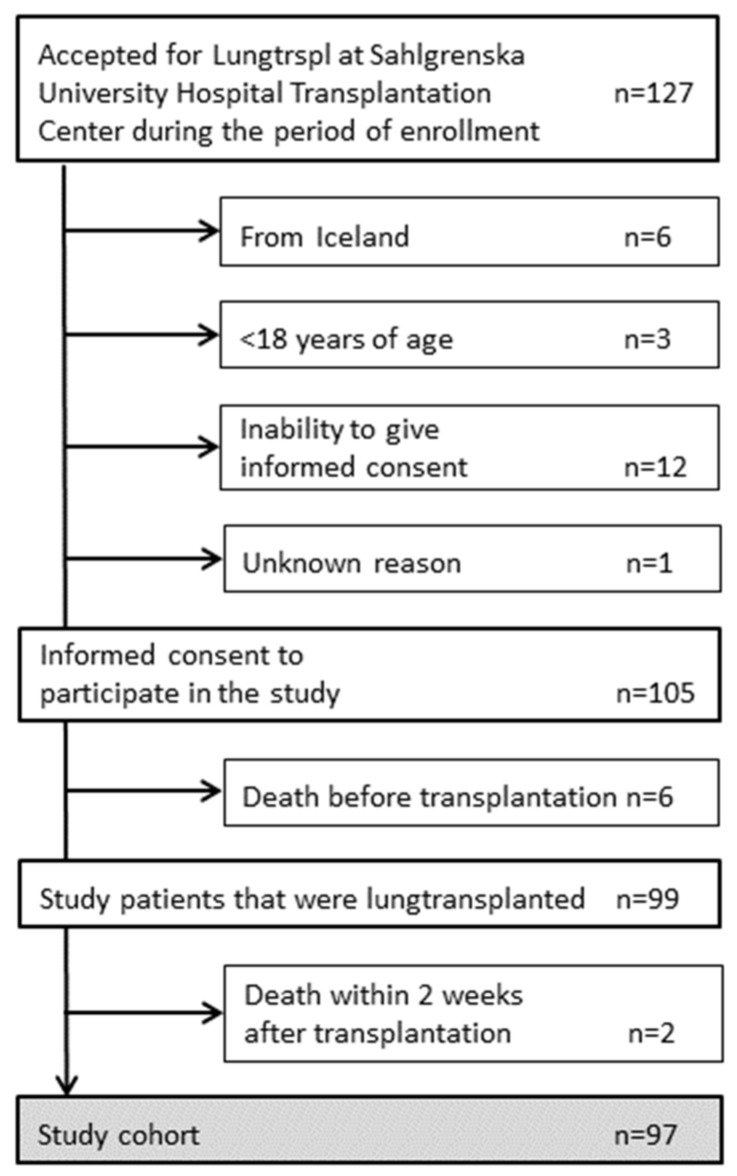
Inclusion of study patients.

**Figure 2:**
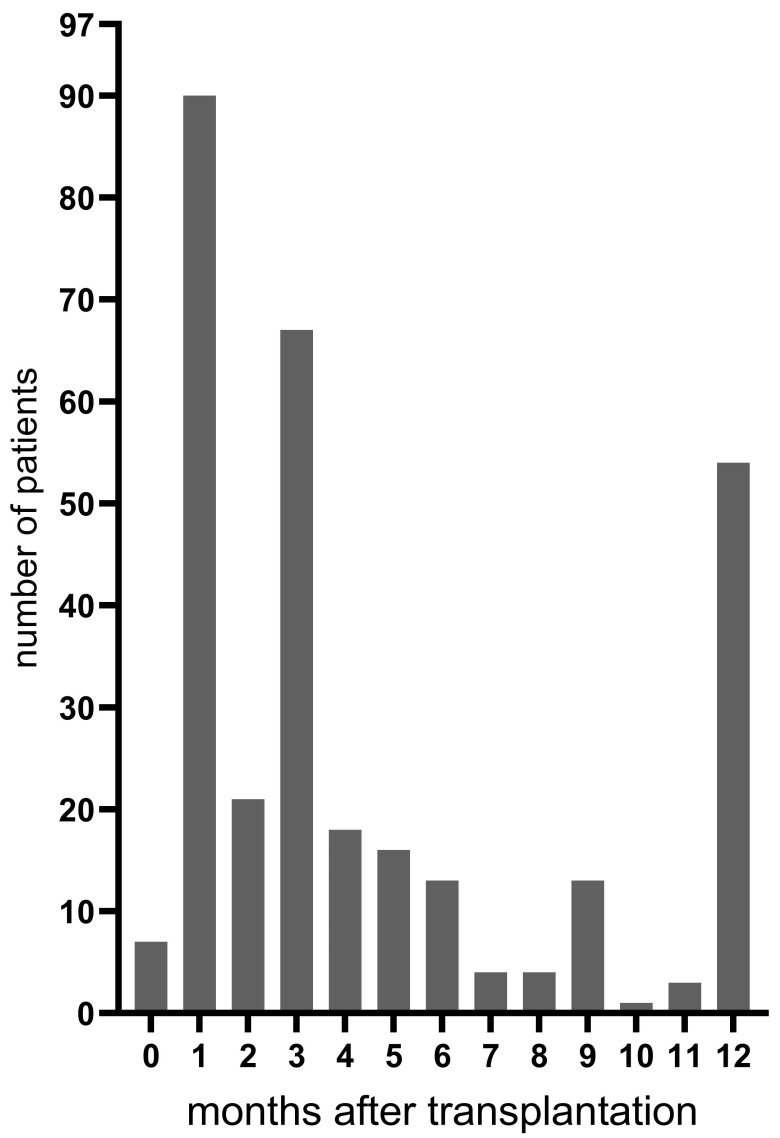
Time points of performed bronchoscopies after transplantation.

A total of 105 eligible patients accepted for lung transplantation at the Sahlgrenska University Hospital gave informed consent to participate in the study. Six patients died while on the waiting list, and two patients died within two weeks after transplantation, resulting in 97 patients included in the study (Figure 1). The clinical characteristics of the study cohort are shown in Table 1. A median and range of 3 (1–10) follow-up bronchoscopies per patient were performed during the first postoperative year. The distribution of bronchoscopies is shown in Figure 2. Thirteen of the included patients (13%) died during the one-year study period, with the majority being between three and twelve months after transplantation. Since no patient had positive pre-transplant cultures yielding *Aspergillus*, all patients received antifungal prophylaxis with oral nystatin only according to clinical routine.

### 3.2. Fungal Tracheobronchitis and Colonization

In total, 61/97 (63%) had *Candida* or *Aspergillus* isolated from BALF at least once during the first year after transplantation (Table 2). There were 22 episodes of *Candida* tracheobronchitis (1 proven and 21 probable) and 15 episodes of *Aspergillus* tracheobronchitis (2 proven and 13 probable) in 31 patients. Six patients had an episode of *Aspergillus* tracheobronchitis following an earlier episode of *Candida* tracheobronchitis. Three patients with *Aspergillus* tracheobronchitis had concomitant signs of invasive *Aspergillus* pneumonia. The cumulative one-year incidence of fungal, *Candida* and *Aspergillus* tracheobronchitis was 33%, 23% and 16%, respectively. After transplantation, *Candida* tracheobronchitis occurred at a median of 0.8 months (range 0.2–12), while *Aspergillus* tracheobronchitis occurred at a median of 3.4 months (range 0.7–9.6). Among the 37 episodes classified as fungal tracheobronchitis, there was concomitant growth of potentially pathogenic bacteria in BALF in 14 episodes (38%): *Pseudomonas aeruginosa* (*n* = 6), *Staphylococcus aureus* (*n* = 4), *Enterococcus* spp. (*n* = 2) and other Gram-negative bacteria (*n* = 2). The histopathologic analysis of transbronchial biopsies showed signs of acute rejection in 4 (11%) out of these 37 episodes of tracheobronchitis. In 10 of the 37 episodes classified as fungal tracheobronchitis, no transbronchial biopsies were performed.

Sixteen and eight patients had at least one culture from BALF with growth of *Candida* or *Aspergillus*, respectively, but without signs of tracheobronchitis, which was defined as colonization. Forty-four patients (45%) had no evidence of fungal colonization or tracheobronchitis during the 12 months of follow-up.

### 3.3. Diagnostic Markers

**Figure 3:**
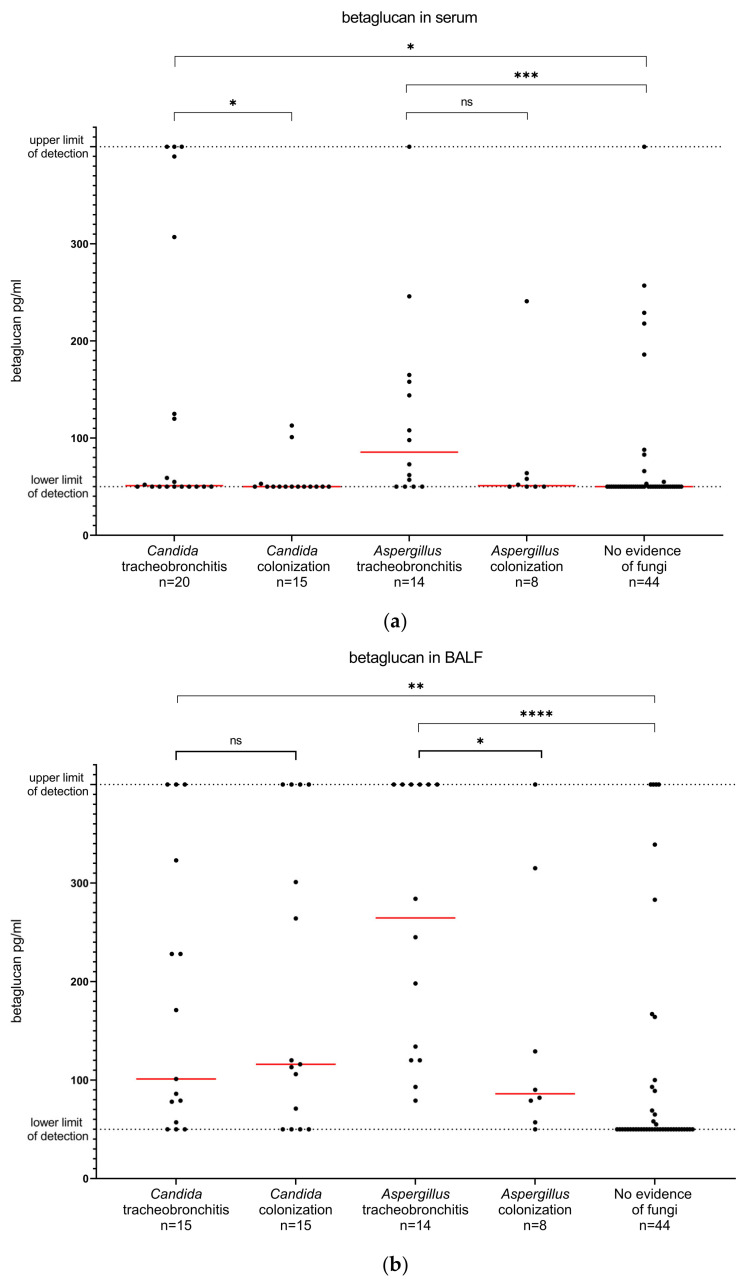

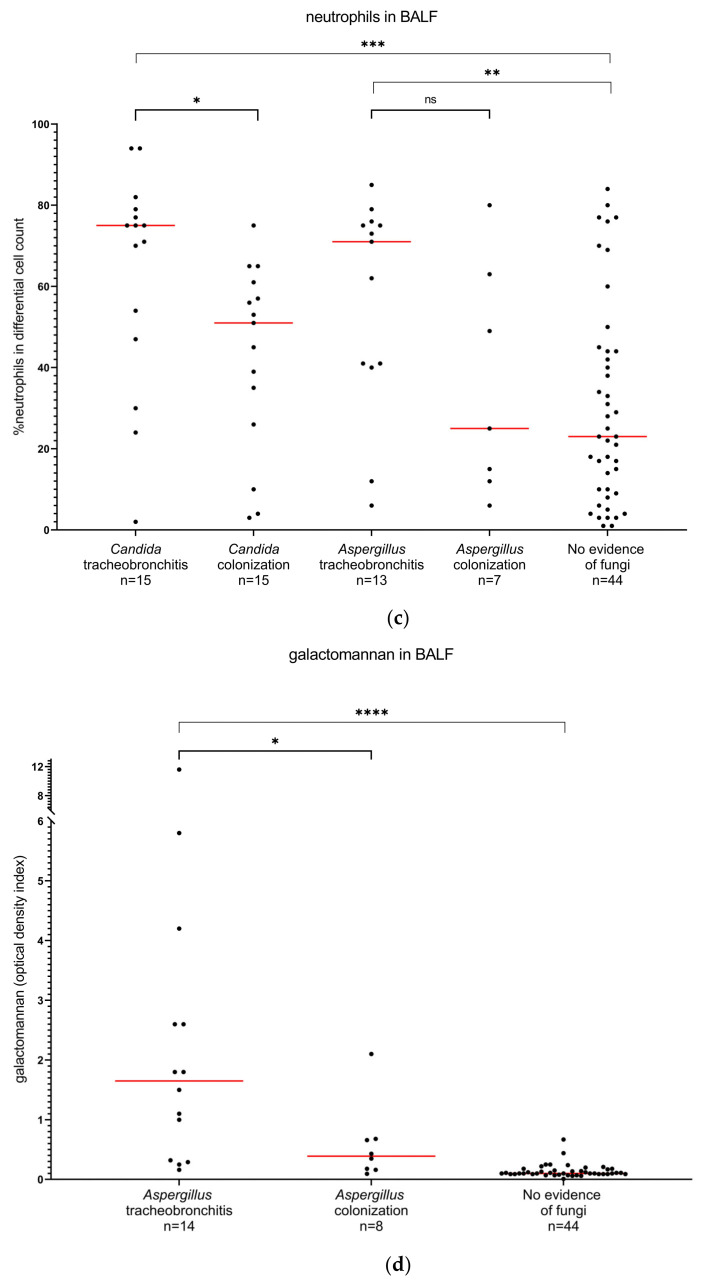
Betaglucan in serum (**a**), betaglucan in BALF (**b**), neutrophils in BALF (**c**) and galactomannan in BALF (**d**) in patients with fungal tracheobronchitis, fungal colonization and no evidence of fungi during the first year after lung transplantation. A *p*-value of <0.05 was considered statistically significant (* *p* < 0.05, ** *p* < 0.01, *** *p* < 0.001 and **** *p* < 0.0001). The red line corresponds to the median value.

The levels of the diagnostic markers in the different patient groups, including the number of analyzed samples for each group, are shown in Figure 3. Although there was no difference in the median serum betaglucan levels between patients with *Candida* tracheobronchitis and patients with *Candida* colonization, there was a significant difference in mean ranks between the two groups (*p* = 0.04). There was a significant difference in the BALF betaglucan levels between patients with *Aspergillus* tracheobronchitis and patients with *Aspergillus* colonization (*p* = 0.03). The median neutrophil counts in BALF were higher in patients with *Candida* tracheobronchitis compared to patients with *Candida* colonization, as well as in patients with *Aspergillus* tracheobronchitis compared to patients with *Aspergillus* colonization. However, the difference in mean ranks was statistically significant only for patients with *Candida* tracheobronchitis and colonization (*p* = 0.02). The median galactomannan index in BALF was higher in patients with *Aspergillus* tracheobronchitis than in patients with *Aspergillus* colonization, and the difference in mean ranks between the groups was statistically significant (*p* = 0.04).

The results of all performed ROC curve analyses are provided as supplementary data (Supplementary Figure S1). Neutrophil count in BALF was the only diagnostic marker that showed an acceptable ability to discriminate (1) *Candida* tracheobronchitis from colonization or (2) *Candida* tracheobronchitis from colonization or no evidence of fungi (area under the ROC curves 0.75 (95% CI 0.57–0.94, *p* = 0.02) and 0.79 (95% CI 0.65–0.93, *p* = 0.005), respectively).

For the discrimination of *Aspergillus* tracheobronchitis from colonization, only betaglucan in BALF had an acceptable area under the ROC curve (0.77 (95% CI 0.55–0.99, *p* = 0.04). However, when comparing *Aspergillus* tracheobronchitis with *Aspergillus* colonization or no evidence of fungi, both betaglucan and galactomannan in BALF showed a high discriminatory ability (area under the ROC curves 0.86 (95% CI 0.78–0.95, *p* < 0.0001) and 0.93 (95% CI 0.85–0.99, *p* < 0.0001), respectively).

The results regarding the sensitivity and specificity of neutrophils in BALF for the diagnosis of *Candida* tracheobronchitis (compared to *Candida* colonization or no evidence of fungi) and betaglucan and galactomannan in BALF for the diagnosis of *Aspergillus* tracheobronchitis (compared to *Aspergillus* colonization or no evidence of fungi) are shown in Table 3.

## 4. Discussion

In this prospective study of 97 lung-transplant recipients at Sweden’s largest center for cardiothoracic organ transplantations, the occurrence of fungi in the lower respiratory tract during the first year after transplantation was high. The one-year incidence of *Candida* tracheobronchitis was higher than that of *Aspergillus* tracheobronchitis. We found that diagnostic markers such as betaglucan and galactomannan in BALF may help to discriminate between *Aspergillus* tracheobronchitis and colonization, and high neutrophil counts in BALF together with a culture yielding *Candida* may be indicative of *Candida* tracheobronchitis.

Fungal tracheobronchitis caused by *Aspergillus*, as well as *Candida* spp., is a recognized infectious complication after lung transplantation, and treatment recommendations for both *Aspergillus* tracheobronchitis and *Candida* tracheobronchitis are included in international guidelines [4,21]. Despite this, there is a paucity of published data on *Candida* tracheobronchitis. The recommended criteria used in clinical studies to define fungal infections in lung-transplant recipients [10,11] include a definition for probable *Aspergillus* tracheobronchitis but not for probable *Candida* tracheobronchitis. Reliable data on the incidence of *Candida* tracheobronchitis after lung transplantation are thus lacking. To the best of our knowledge, this is the first study reporting the incidence of fungal tracheobronchitis after lung transplantation, including tracheobronchial infections caused by *Candida* spp. Previous studies report one-year incidences of invasive fungal infections after lung transplantation of 3–14% [1,2,4]. Another recent study reports a 19% prevalence rate of invasive fungal infections during the first 180 days after lung transplantation [3]. Our study focused on tracheobronchitis only and did not include other invasive fungal infections; however, the one-year incidence found in our cohort was higher (33%) than in these previous reports. One explanation for this discrepancy might be the lack of use of systemic antifungal prophylaxis at our center. However, in a previous study reporting the overall microbiologic findings in BALF among lung transplant recipients in two Swedish transplant centers [13], we found no increased prevalence of fungal colonization or infection in our center compared to the other center where systemic fluconazole was given as antifungal prophylaxis (unpublished data). Another explanation for the higher incidence found in our study may be an underestimation of the prevalence of *Candida* tracheobronchitis in previous studies, considering that established definitions only allow for inclusion of proven cases of *Candida* tracheobronchitis. However, we are aware of the limitations associated with the inclusion of probable cases of infection based on a non-standardized case definition, and future studies are warranted to confirm our findings regarding the high incidence of *Candida* tracheobronchitis after lung transplantation.

Fungal tracheobronchitis after lung transplantation is associated with bronchial complications [22] and excess mortality [6,23,24], which calls for early antifungal treatment. However, diagnosis of fungal tracheobronchitis may be challenging due to the low sensitivity of fungal cultures, in particular regarding molds such as *Aspergillus* spp. Diagnostic markers that could help diagnose fungal tracheobronchitis in the absence of a positive fungal culture would thus be of great value. On the other hand, in the transplanted population, treatment with antifungals may cause severe adverse events, as well as drug-drug interactions, and unnecessary antifungal treatment should be avoided. During the follow-up period of 12 months, *Candida* and *Aspergillus* spp. were isolated from BALF in 39% and 24% of all patients, respectively. Other studies report similar rates of *Aspergillus* colonization [4,24,25] and even higher rates of *Candida* colonization [14,26]. Considering the high frequency of fungal isolation from BALF in lung-transplant recipients during the first postoperative year and the sometimes unspecific endobronchial lesions found at bronchoscopy, there is a need for diagnostic markers that can help to differentiate fungal tracheobronchitis from colonization and aid in the decision to treat or not to treat [27].

We found that high neutrophil counts in BALF could help to discriminate *Candida* tracheobronchitis from *Candida* colonization, and neutrophils in BALF could be a complementary tool for the diagnosis of *Candida* tracheobronchitis. High neutrophil counts in BALF after lung transplantation can also be seen in acute lung allograft rejection and infectious conditions other than fungal infection [16,28], but our results showed that a cutoff for neutrophil proportion in BALF of >75% had a high specificity (92%) for the diagnosis of *Candida* tracheobronchitis. The sensitivity, however, was very low, and a positive *Candida* culture in BALF, together with low neutrophil counts, does not rule out the possibility of a diagnosis of *Candida* tracheobronchitis.

The available commercial betaglucan assays are recommended for serum samples; however, several previous studies [17,29,30,31,32,33,34], including one meta-analysis [18], evaluated the performance of betaglucan in BALF samples for the diagnosis of invasive pulmonary fungal infections in different patient groups with varying results. In our study, serum betaglucan levels could not discriminate patients with fungal tracheobronchitis from those with fungal colonization or no evidence of fungi. Thus, our results do not support the use of betaglucan measurement in serum for the diagnosis of fungal tracheobronchitis in lung-transplant recipients. This finding is in line with two other prospective studies showing a low sensitivity and specificity of serum-betaglucan levels for the diagnosis of invasive fungal disease after lung transplantation [34,35]. On the other hand, the results of betaglucan levels in BALF in our study were more promising. Among patients with *Aspergillus*, betaglucan levels in BALF were significantly higher in patients with tracheobronchitis than in patients with colonization, and the ability of betaglucan in BALF to distinguish patients with *Aspergillus* tracheobronchitis from patients with colonization or no evidence of fungi was relatively high (area under the ROC curve of 0.86). The sensitivity and specificity of betaglucan in BALF for the diagnosis of *Aspergillus* tracheobronchitis, at a cutoff level of 110 pg/mL, was moderate (86% and 79%, respectively); however, at a higher cutoff level of 370 pg/mL, betaglucan in BALF could correctly identify 90% of all patients with *Aspergillus* tracheobronchitis, but at the expense of a low sensitivity. In contrast, betaglucan in BALF was not useful for the diagnosis of *Candida* tracheobronchitis. To the best of our knowledge, our study is the first to evaluate betaglucan in BALF specifically for the diagnosis of fungal tracheobronchitis in lung-transplant recipients. Two previous studies evaluating betaglucan in BALF for the diagnosis of invasive pulmonary aspergillosis in solid-organ-transplant [34] and lung-transplant [17] recipients show a sensitivity of 60–79% and a specificity of 39–70% at a cutoff level of approximately 100 pg/mL. The diagnostic performance of betaglucan in our study was thus higher, which could imply that betaglucan in BALF may be more useful for the diagnosis of *Aspergillus* tracheobronchitis than for pulmonary aspergillosis.

The measurement of galactomannan in BALF is a well-established and recommended method for the diagnosis of pulmonary aspergillosis in lung-transplant recipients [4,12,36]. However, data on the utility of this diagnostic tool for diagnosing *Aspergillus* tracheobronchitis and for the differentiation of tracheobronchitis from colonization are scarce. We found significantly higher galactomannan levels in BALF in patients with *Aspergillus* tracheobronchitis than in patients with only colonization. These findings align with previous studies comparing galactomannan indices in BALF in lung-transplant recipients with invasive pulmonary aspergillosis and *Aspergillus* colonization [37,38,39], although few cases of tracheobronchitis were included in those studies. The assessment of the overall performance of galactomannan in BALF for the diagnosis of *Aspergillus* tracheobronchitis showed a very high area under the ROC curve (0.93), with >90% specificity at cutoff levels >0.5. Galactomannan in BALF thus seems to be a useful tool not only for the diagnosis of invasive *Aspergillus* pneumonia but also for the diagnosis of *Aspergillus* tracheobronchitis.

Our study has several limitations. As already mentioned, one major limitation is the use of a non-standardized definition for *Candida* tracheobronchitis. However, while waiting for a consensus definition for probable *Candida* tracheobronchitis in lung-transplant recipients to be published for use in future clinical studies, our attempt to address this important condition by adopting the consensus definitions of *Aspergillus* tracheobronchitis in a wider context of fungal origin may be acceptable and perhaps the most reasonable approach to be used at this point. Hence, our study contributes new data regarding the underreported condition of *Candida* tracheobronchitis and adds valuable information to the literature on infections in lung-transplant recipients.

Similar to the majority of other studies on this subject, our study predominantly included probable rather than proven cases of fungal tracheobronchitis, where macroscopic bronchoscopic findings, along with microbiologic criteria, are decisive for the case classification [10]. A limitation inherent to this classification is the difficulty in deciding the true causative organism of the tracheobronchial lesions in patients with concurrent growth of both fungi and bacteria in BALF. Nevertheless, a minority of the patients classified as fungal tracheobronchitis in our study had coexisting positive bacterial cultures. A large overestimation of the number of cases of fungal tracheobronchitis thus seems unlikely.

Another limitation is the somewhat limited size of the studied cohort, which resulted in wide confidence intervals for the results of comparative analyses. It also precluded a further sub-analysis of predictive values of the fungal markers at the different time points of clinical control during the first year after lung transplantation. Finally, the single-center design of this study warrants a certain level of caution regarding the generalization of the results.

## 5. Conclusions

We found a high one-year incidence of fungal tracheobronchitis in our lung-transplanted cohort, where *Candida* was the dominating species. A standardized case definition for probable *Candida* tracheobronchitis is yet missing, and in the light of the results of our study, concern is raised regarding a possible underestimation of the true burden of fungal tracheobronchitis in previous epidemiologic studies on lung-transplant patients. However, the clinical relevance of fungal growth in BALF after lung transplantation is ambiguous. Our results suggest that diagnostic markers in BALF, such as neutrophils, betaglucan and galactomannan, may help to discriminate fungal colonization from tracheobronchitis. Further prospective studies assessing the predictive values of these diagnostic markers for fungal tracheobronchitis and their potential role as tools for surveillance of fungal infection after lung transplantation are warranted.

## Figures and Tables

**Table 1:** Characteristics of the study cohort.

	*n* = 97
Male, *n* (%)	45 (46)
Recipient age, years median (range)	59 (18–70)
Donor age, years median (range)	54 (14–76)
Type of lung transplantation, *n* (%)	
Single	7 (7)
Double	82 (85)
Re-transplantation	8 (8)
Cold ischemia time, hours median (range) ^a^	7.1 (1.8–13.8)
Pretransplant underlying condition, *n* (%)	
Chronic obstructive pulmonary disease	29 (30)
Pulmonary fibrosis	25 (26)
Cystic fibrosis	11 (11)
Alpha-1 antitrypsin deficiency	12 (13)
Pulmonary arterial hypertension	4 (4)
Bronchiolitis obliterans syndrome	7 (7)
Sarcoidosis	1 (1)
Bronchiectasis	1 (1)
Other ^b^	7 (7)
Calcineurin inhibitor during the first postoperative year ^c^, *n* (%)	
Cyclosporine	78 (80)
Tacrolimus	19 (20)
Bronchoscopies per patient during the first postoperative year, *n* median (range)	3 (1–10)
Death during the first postoperative year, *n* (%)	13 (13)
within one month after transplantation ^d^	1
one to three months after transplantation	2
three to twelve months after transplantation	10

^a^ For double lung transplantation, the longest ischemia time was registered. The cold ischemia time from 13 ex vivo lung perfusion grafts was excluded. ^b^ Systemic lupus erythematosus (*n* = 1), Langerhans histiocytosis (*n* = 1), pulmonary embolism (*n* = 1), Kartagener Syndrome (*n* = 1), lymphangioleiomyomatosis (*n* = 2), and dermatomyositis (*n* = 1).^c^ Five patients switched to another calcineurin inhibitor 6–9 months after transplantation. ^d^ Two patients who died within two weeks from transplantation were not included in this study.

**Table 2:** Fungal colonization and tracheobronchitis.

Total number of patients, *n*	97
*Candida*	
Colonization only, *n* (%)	16 (16)
Time after transplantation ^a^, median months (range)	0.8 (0.6–3.1)
Tracheobronchitis, *n* (%)	22 (23)
Time after transplantation ^b^, median months (range)	0.8 (0.2–12)
*Aspergillus*	
Colonization only, *n* (%)	8 (8)
Time after transplantation ^b^, median months (range)	3 (0.8–12.0)
Tracheobronchitis, *n* (%)	15 (15)
Time after transplantation, median months (range)	3.4 (0.7–9.6)
No evidence of fungal colonization nor tracheobronchitis, *n* (%)	44 (45)

^a^ Referring to the first episode of BALF culture yielding fungi. ^b^ Referring to the first episode when criteria for tracheobronchitis were fulfilled.

**Table 3:** Sensitivity and specificity of diagnostic markers for the diagnosis of *Candida* and *Aspergillus* tracheobronchitis. Controls consist of patients with *Candida* and *Aspergillus* colonization or patients with no evidence of fungi in BALF, respectively.

Diagnostic Markers in BALF	Sensitivity, % (95% CI)	Specificity, % (95% CI)
*Candida* tracheobronchitis		
neutrophils > 75%	33.3 (15.2–58.3)	91.5 (81.7–96.3)
*Aspergillus* tracheobronchitis		
betaglucan > 110 pg/mL	85.7 (60.1–97.5)	78.8 (66.0–87.8)
betaglucan > 370 pg/mL	42.9 (21.4–67.4)	90.4 (79.4–95.8)
galactomannan > 0.25	90.9 (62.2–99.5)	82.7 (70.3–90.6)
galactomannan > 0.5	63.6 (35.4–84.8)	90.4 (79.4–95.8)
galactomannan > 1.0	63.6 (35.4–84.8)	98.1 (89.9–99.9)
neutrophils > 75%	23.1 (8.2–50.3)	88.2 (76.6–94.5)
betaglucan > 110 pg/mL AND galactomannan > 0.5	54.6 (23.4–83.3)	96.2 (86.8–99.5)
betaglucan > 110 pg/mL OR galactomannan > 0.5	100 (71.5–100)	75 (61.1–86)

For the evaluation of galactomannan, three patients with galactomannan as the only mycological criterion for *Aspergillus* tracheobronchitis were excluded.

## Data Availability

The data presented in this study are available from the corresponding author upon request. The data are not publicly available due to ethical restrictions.

## Supplementary Materials

The following supporting information can be downloaded at https://www.mdpi.com/article/10.3390/jof9010003/s1. Figure S1: ROC curve analyses of the different diagnostic markers.
