# Supplementary material for: Fungal Tracheobronchitis in Lung Transplant Recipients: Incidence and Utility of Diagnostic Markers
Source: J Fungi (Basel). 2022 Dec 20;9(1):3. doi: 10.3390/jof9010003 (PMC9861951; doi:10.3390/jof9010003)
Supplement: Supplementary file 1 [file jof-09-00003-s001.zip › jof-2092820-supplementary.pdf]

Figure S1. ROC curve analyses to assess the ability of the different diagnostic markers to discriminate (A) *Candida* tracheobronchitis from colonization, (B) *Candida* tracheobronchitis from colonization or no evidence of fungi, (C) *Aspergillus* tracheobronchitis from colonization and (D) *Aspergillus* tracheobronchitis from colonization or no evidence of fungi.

A

Betaglucan in serum
Candida tracheobronchitis vs colonization

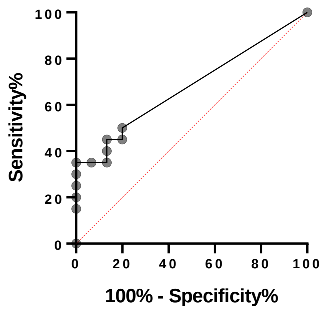

Area under the ROC curve	
Area	0.6767
Std. Error	0.09010
95% confidence interval	0.5001 to 0.8533
P value	0.0773
Data	
Controls (*Candida* colonization n=15)	15
Patients (*Candida* tracheobronchitis n=20)	20

Betaglucan in BALF
Candida tracheobronchitis vs colonization

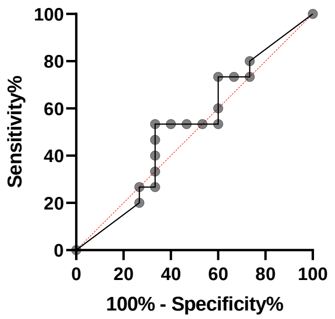

Area under the ROC curve	
Area	0.5244
Std. Error	0.1081
95% confidence interval	0.3126 to 0.7363
P value	0.8195
Data	
Controls (*Candida* colonization n=15)	15
Patients (*Candida* tracheobronchitis n=15)	15

Neutrophils in BALF
Candida tracheobronchitis vs colonization

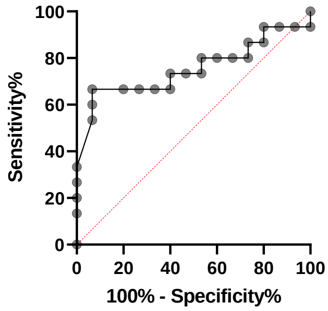

Area under the ROC curve	
Area	0.7533
Std. Error	0.09515
95% confidence interval	0.5668 to 0.9398
P value	0.0181
Data	
Controls (*Candida* colonization n=15)	15
Patients (*Candida* tracheobronchitis n=15)	15

B

Betaglucan in serum
Candida tracheobronchitis vs
colonization or no evidence of fungi

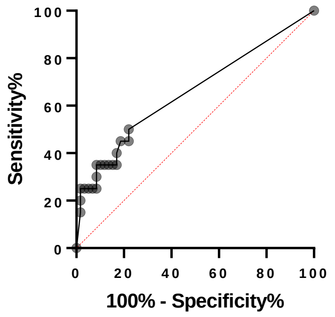

Area under the ROC curve	
Area	0.655
Std. Error	0.0765
95% confidence interval	0.505 to 0.805
P value	0.0391
Data	
Controls (CandCol+NoFungi)	59
Patients (*Candida* tracheobronchitis n=20)	20

Betaglucan in BALF
Candida tracheobronchitis vs
colonization or no evidence of fungi

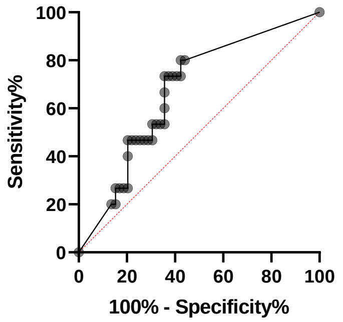

Area under the ROC curve	
Area	0.6718
Std. Error	0.07341
95% confidence interval	0.5279 to 0.8156
P value	0.0410
Data	
Controls (CandCol+NoFungi)	59
Patients (*Candida* tracheobronchitis n=15)	15

Neutrophils in BALF
Candida tracheobronchitis vs
colonization or no evidence of fungi

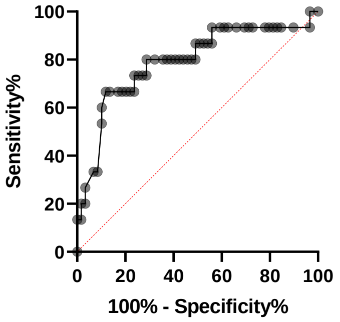

Area under the ROC curve	
Area	0.7910
Std. Error	0.07274
95% confidence interval	0.6484 to 0.9335
P value	0.0005
Data	
Controls (CandCol+NoFungi)	59
Patients (*Candida* tracheobronchitis n=15)	15

C

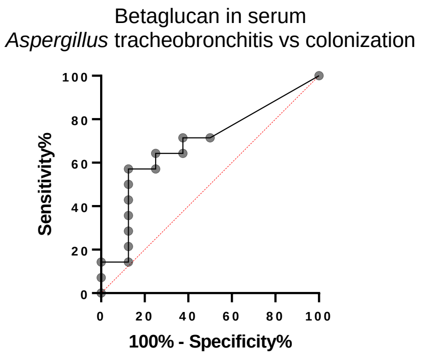

Area under the ROC curve	
Area	0.6875
Std. Error	0.1190
95% confidence interval	0.4543 to 0.9207
P value	0.1518
Data	
Controls (*Aspergillus* colonization n=8)	8
Patients (*Aspergillus* tracheobronchitis n=14)	14

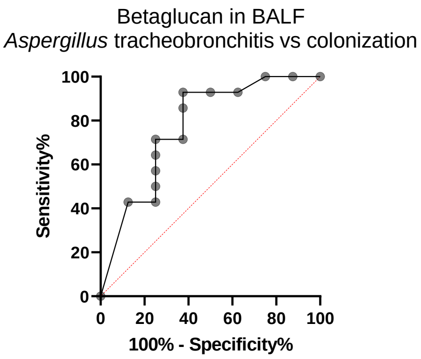

Area under the ROC curve	
Area	0.7723
Std. Error	0.1133
95% confidence interval	0.5502 to 0.9944
P value	0.0374
Data	
Controls (*Aspergillus* colonization n=8)	8
Patients (*Aspergillus* tracheobronchitis n=14)	14

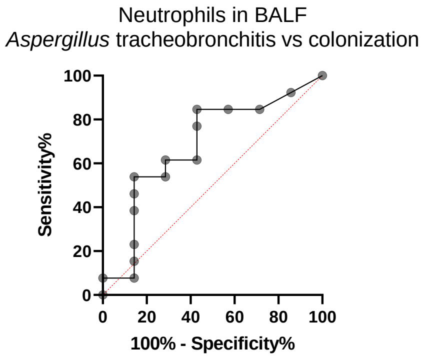

Area under the ROC curve	
Area	0.6813
Std. Error	0.1330
95% confidence interval	0.4206 to 0.9420
P value	0.1910
Data	
Controls (*Aspergillus* colonization n=7)	7
Patients (*Aspergillus* tracheobronchitis n=13)	13

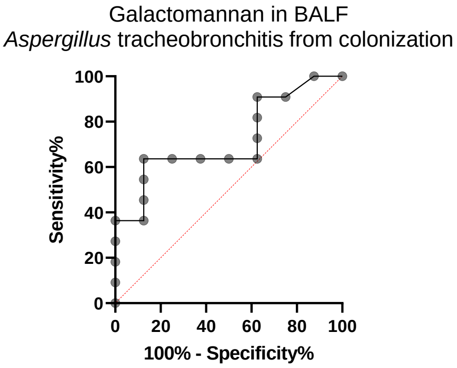

Area under the ROC curve	
Area	0.7216
Std. Error	0.1197
95% confidence interval	0.4870 to 0.9562
P value	0.1074
Data	
Controls (*Aspergillus* colonization n=8)	8
Patients (*Aspergillus* tracheobronchitis n=11)	11

D

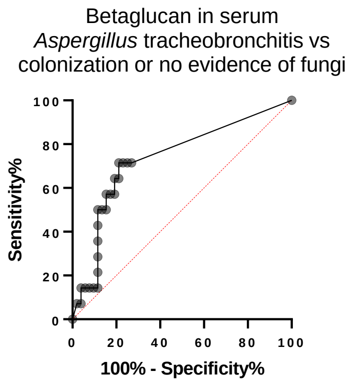

Area under the ROC curve	
Area	0.7342
Std. Error	0.07927
95% confidence interval	0.5788 to 0.8896
P value	0.0075
Data	
Controls (AspCol+NoFungi)	52
Patients (*Aspergillus* tracheobronchitis n=14)	14

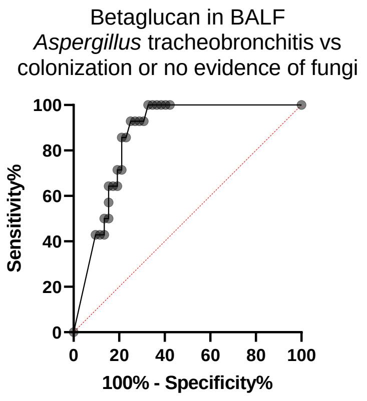

Area under the ROC curve	
Area	0.864
Std. Error	0.0437
95% confidence interval	0.778 to 0.950
P value	<0.0001
Data	
Controls (AspCol+NoFungi)	52
Patients (*Aspergillus* tracheobronchitis n=14)	14

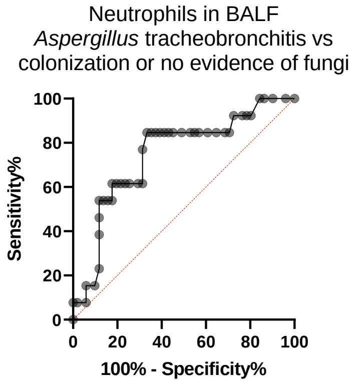

Area under the ROC curve	
Area	0.7459
Std. Error	0.07694
95% confidence interval	0.5950 to 0.8967
P value	0.0065
Data	
Controls (AspCol+NoFungi)	51
Patients (*Aspergillus* tracheobronchitis n=13)	13

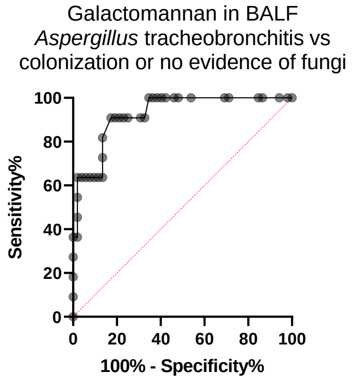

Area under the ROC curve	
Area	0.9257
Std. Error	0.03721
95% confidence interval	0.8528 to 0.9986
P value	<0.0001
Data	
Controls (AspCol+NoFungi)	52
Patients (*Aspergillus* tracheobronchitis n=11)	11
